# Bioconversion of Different Waste Streams of Animal and Vegetal Origin and Manure by Black Soldier Fly Larvae *Hermetia illucens* L. (Diptera: Stratiomyidae)

**DOI:** 10.3390/insects14020204

**Published:** 2023-02-17

**Authors:** Somaya Naser El Deen, Klaas van Rozen, Hellen Elissen, Piet van Wikselaar, Istvan Fodor, Rommie van der Weide, Elise Federica Hoek-van den Hil, Arya Rezaei Far, Teun Veldkamp

**Affiliations:** 1 Wageningen Livestock Research, De Elst 1, 6700 AH Wageningen, The Netherlands; 2 Wageningen Plant Research, Edelhertweg 1, 8219 PH Lelystad, The Netherlands; 3 Wageningen Food Safety Research, Akkermaalsbos 2, 6708 WB Wageningen, The Netherlands

**Keywords:** fast food waste, fatty acids, frass, macronutrients

## Abstract

**Simple Summary:**

The worldwide waste problem has large environmental impacts and huge efforts have been made to mitigate this problem. Black soldier fly (*Hermetia illucens*) larvae (BSFL) were proposed as an efficient way to get rid of organic waste, acting by converting the waste into a protein- and lipid-rich biomass suitable for various purposes such as animal feed, biodiesel and chitin. This article studies larval growth and development and waste reduction and conversion indexes of BSFL when reared on six different waste streams and a control substrate (chicken feed): pig manure slurry mixed with roadside silage grass, the organic wet fraction of municipal household waste, secondary sludge from slaughter waste, fast food waste, mushroom stems and pig manure solids. In addition, the larval fatty acid profile and the chemical composition of the frass were also analyzed. The larval growth as well as the waste reduction index and the efficiency of conversion of ingested substrate were highest in the fast food waste (FFW) substrate. The larvae reared on FFW had high fat content and produced fat-rich frass with high dry matter content. Slaughter waste, pig manure and mushroom stems also showed good potential for bioconversion and led to protein-rich larvae.

**Abstract:**

Black soldier fly larvae (BSFL) are considered a commercially viable solution for global organic waste problems. The objective of this study was to assess the feasibility of rearing BSFL on a wide range of low-value waste streams and its potential to transform them into high-quality animal feed and fertilizer. Six waste streams of different origins were selected and each tested in triplicate. Several parameters were analysed: growth performance, waste reduction index (WRI), conversion efficiency (ECI) and larval composition. Frass composition was also analysed. Larvae reared on fast food waste (FFW) had the highest ECI and WRI and the lowest values when reared on pig manure slurry mixed with silage grass (PMLSG) and slaughter waste (SW). The highest protein content was found for larvae reared on mushroom stems (MS) although this substrate had the lowest protein content. Moreover, the frass nutritional profile was proportionally related to the substrate’s nutritional profile: the protein-rich substrate (SW) resulted in protein-rich frass and the low-protein substrate (MS) resulted in protein-poor frass. The same was true for the lipid content. In conclusion, this study showed that BSFL can be successfully reared on a wide range of waste streams that can affect the larval and frass chemical compositions.

## 1. Introduction

Wasted food, hunger, the expanding population and urbanization are all well-known global problems threatening food security. Agriculture, agro-industrial processes, restaurants and households generate a high amount of organic by-products and waste. The food waste index report estimated that around 931 million tons of food waste were generated in 2019, meaning that 17% of the total global food production is wasted (61% in households, 26% in food service and 13% in retail) [1]. The EU’s Farm to Fork strategy aims to reduce food losses and waste across the EU by 2023 as an important part of the strategy [2]. Similarly, one of the Sustainable Development Goals (SDGs) of the United Nations is to halve the global food waste of both retail and consumer levels per capita and to reduce food loss throughout the production and supply chains by 2030 [3]. Several innovative strategies were implemented to tackle the food waste problem and the core of these strategies is based on the three Rs rule: reduce, reuse and recycle. Rearing insects can play a significant role in the reuse and recycle strategies [4]. Along with the food waste and loss problems, the rapidly growing human population, with an increasing demand for animal-derived protein, is another hurdle facing food security that is leading to the overuse of the available natural resources [5]. The United Nations mentioned that the current world population of 7.6 billion is expected to reach 8.6 billion in 2030 and 9.8 billion in 2050 [6]. The increasing population and thus consumption levels are placing unprecedented demands on agriculture and natural resources, even the annual rise in yield cannot compensate for the wealthy-becoming population [7]. In fact, global meat production including poultry meat, pig meat and beef is expected to rise up to 373 million tons by 2030, causing high environmental implications such as greenhouse gas emissions, water consumption, land use and other impacts [8]. Therefore, innovations are needed to ensure food security while reducing the environmental impacts of food production.

Insect-based bioconversion is one of the proposed solutions for the above-mentioned problems and concerns that have lately drawn a lot of attention for several reasons [4,9,10]. Rearing insects can potentially bring back lost nutrients into the food chain in the form of protein-rich animal feed, human food and fertilizer [4]. Insects have proven their ability for treating biological wastes and being relatively environmentally friendly and cost-efficient [11]. *Hermetia illucens* L., also known as the black soldier fly (BSF), is the most commonly used and suitable species for mass production and has several agricultural and industrial applications [10,12]. It has a short life cycle and its larvae are characterized by a low feed conversion ratio [4,11]. BSFL are able to convert and recover nutrients from a vast variety of materials such as wastes from abattoirs [13], food [14], fruits and vegetables [15,16], and the feces of humans and livestock manure [17] into valuable protein-rich and fat-rich larval biomass [11,12,18]. Furthermore, the nutritional profile of BSFL is comparable to several oilseeds including hempseed [19], flaxseed [20] and rapeseed [20] with up to 28% of protein and 40% of oil content [21]. The larval biomass could (partially) meet the nutritional needs of fish, poultry [11], pets and pigs and is increasingly applied in multiple industries for animal feeding, biodiesel production, biopolymers (chitin) and soil composting [11,22].

BSFL show an optimal bioconversion efficiency under ideal environmental conditions in terms of humidity, temperature, oxygen level and physical and nutritional profiles of the substrates [11,22]. Although the BSFL are nonselective in terms of their rearing substrates [12], variabilities in chemical and physical characteristics of the substrates can highly affect growth rates, the development time and bioconversion activities of BSFL [23,24]. In addition, the type of substrate significantly affected the developmental rate and nutritional composition of insects [4,14]. The nutritional composition of BSFL may vary with different substrates and under different rearing conditions. The protein content of BSFL ranges between 35 and 57%, and the fat content between 15 and 49% on a dry matter basis [12,25,26,27]. Similarly, the larval fatty acid profile may vary based on different substrates [14,28,29] while the amino acid profile was commonly known to be more stable [14,30]. However, new studies proved that the substrate can partially affect the amino acid profile [18,31].

Moreover, BSFL can endure liquid substrates including wet wastes with up to 70–75% moisture which allows them to decompose a large range of waste streams. This is evident with promising results in studies rearing BSFL on high-moisture substrates such as restaurant and kitchen wastes [4,32,33], pig manure and chicken litter [33,34,35].

Therefore, in the current study pig manure, roadside silage sludge, the organic wet fraction of municipal household waste, secondary sludge from slaughter waste, fast food waste and mushroom stems were tested in comparison with chicken feed as a control diet. These waste stream products have different dry matter contents and physical characteristics and were selected based on their economic value, availability and high environmental impact. The volume of mushroom stem produced in the Netherlands is large (internal data) and always available, rather than being seasonally constrained, which makes it an attractive substrate for BSFL. Notably, mushroom stems and roadside silage waste streams have not been sufficiently explored and their potentiality as a BSF feeding substrate is still vague. However, although pig manure and slaughter wastes are more researched waste streams they still engender several knowledge gaps. Fast food and organic wet waste are produced at a high level and continuously from restaurants, canteens and homes (internal data) and have a rich nutrient composition which make them suitable as BSFL feeding substrates. In addition to the economic aspect, managing the mentioned waste streams to reduce waste and produce a high quality product also has a positive environmental impact. The objectives are to assess the feasibility of using these waste streams as substrates to grow BSFL in terms of the larval growth rate, waste reduction index and efficiency of conversion of the ingested substrate. Besides the chemical composition and the fatty acid profile of the larvae, the chemical composition of the frass and the larval crude protein and fat masses, conversions and losses were analyzed on the different substrates.

BSFL converting these substrates efficiently into high-grade products would contribute to the circular economy by reducing and/or reusing organic waste. Based on the results of this study, promising waste streams can possibly be selected for further follow-up experiments.

## 2. Materials and Methods

### 2.1. Experimental Set-Up

Black soldier fly larvae (BSFL; *Hermetia illucens*) were obtained from the commercial BSF producing company Bestico, (Berkel en Rodenrijs, The Netherlands). The following substrates were tested: chicken feed (CF; control diet), pig manure slurry mixed with roadside silage grass (PMLSG), the organic wet fraction of municipal household waste (OWF), secondary sludge from slaughter waste (SW), fast food waste (FFW), mushroom stems (MS) and pig manure solid (PMS). Chicken feed is a commercial broiler feed which was used as the control diet [33,36,37]. Pig manure slurry was a mixture of pig feces and urine, and it was mixed with roadside silage grass (1:1 *w*/*w*) to produce PMLSG. The organic wet fraction used in this experiment makes around 30–35% of the municipal household waste. It was contaminated by physical contaminants such as glass and plastic that were not removed. The solid phase of the secondary sludge from slaughter waste was also used as an experimental substrate. The fast food waste consisted of fries, vegetables, bread and meat products but not any non-food waste and was collected within maximum 4 days after disposal. The mushroom stems are a soft substrate and may have been contaminated by soil. The different substrates were selected based on the results of a prior study published by Veldkamp [33].

Substrates were obtained one week before the start of the rearing cycle and stored at 4 °C until use. Some of the substrates were pre-treated in a cutter to decrease particle size which included PMLSG (~2 cm), FFW (~1 cm) and MS substrates (~0.5 cm). All substrates are brought to 35% dry matter by adding water and/or cellulose/wood shavings to decrease or increase the DM in the substrate, respectively. Since the used substrates all have a different weight-to-volume ratio, different quantities of substrates and larvae were added to the containers to maintain a substrate layer of approximately 5 cm such that every BSFL gets 0.54 g of the wet substrate (Table 1). The containers were filled one day before starting the experiment, thus allowing them to adapt to the ambient temperature in the climate chamber without any external heating. On top of each substrate, 1850 starter BSFL (8 day old) per kilogram of wet substrate were incubated in 21 plastic containers (75 cm × 47 cm × 15 cm). Each substrate was tested in triplicate in a climate chamber (7 treatments × 3 replicates). The chamber temperature was set to 28 °C and the relative humidity (RH) was 70% from day 0 until day 5 and was 40–60% from day 6 until the end of the experiment. The rearing chamber was dark. The plastic containers were stacked in three columns each with seven containers (one container per repetition) arranged based on escaping probability, i.e., the containers with the highest moisture content were placed at the bottom to avoid escaping larvae falling into containers below them. Each column was placed in a non-escape box (cubic box; 120 cm × 100 cm × 60 cm). The experimental period was 7 to 8 days which was determined by visual checking of the substrate consumption or the presence of ~10% prepupae.

### 2.2. Measured Parameters

The mean individual start weight of the larvae (5.8 mg) was determined from three samples of, ca., 400 larvae. Before the experiment, triplicate samples of the substrate (~0.5 kg) per repetition (nine samples per treatment) were collected for chemical analysis. An additional two samples (~0.5 kg/sample) per substrate were collected for DM determination.

At the end of the experiment, triplicate samples of the residual substrate/frass and larvae/prepupae were collected from each container for further chemical analysis, and larvae were separated from substrate and frass, counted and weighed.

Dry matter (DM) content of the substrates at the start of the experimental period and the residual substrate/frass fractions and BSFL at the end of the experiment were determined by oven-drying for 48 h at 105 °C

The chemical composition (moisture/water content, crude ash, crude protein (N × 6.25), total fat and crude fiber) of the substrates, larvae and frass was analyzed in AGROLAB LUFA GmbH (Kiel, Germany) according to the methods of sampling and analysis for the official control of feed mentioned in the Commission Regulation (EC) No 152/2009 of 27 January 2009 (https://eur-lex.europa.eu/legal-content/EN/TXT/PDF/?uri=CELEX:32009R0152&from=EN, accessed on 21 January 2023). The fatty acid profile of the larvae was analyzed following the protocols accredited by the German Accreditation Body (https://cdnmedia.eurofins.com/eurofins-germany/media/2728/eurofins_analytik_accreditation_17025_annex_en.pdf, accessed on 21 January 2023).

The average larval growth rate (mg/d) was calculated by the following equation:

GR_larvae_ (mg/d) = (LW_f_ − LW_i_)/d (1)

To evaluate larval efficiency in consuming and metabolizing the growing substrates, the total final biomass (larvae + prepupae) and the residual substrates were weighed. The waste reduction index (WRI) describes the larval ability to reduce substrates, taking into account the number of days the larvae fed on the substrates; therefore, higher values denote a greater ability to reduce the organic matter. The conversion efficiencies are based on DM since considerable variation is present in the DM contents of the substrates.

The waste reduction index and the efficiency of the conversion of the ingested feed (ECI) were calculated for the determination of waste consumed by the larvae and the conversion efficiency of the substrates into insect biomass. The following indexes were calculated:

WRI = ((S_i_ − Fs)/S_i_)/d (2)

ECI = LW_gain_/(S_i_ − Fs) (3)

CP_f larvae_ (g) = LW_f_ × CP_larvae_ (4)

G_f larvae_ (g) = LW_f_ × G_larvae_ (5)

DM_conversion_ (%) = LW_gain_/S_i_ × 100 (6)

CP_conversion_ (%) = CP_larval gain_/CP_substrate_ × 100 (7)

G_conversion_ (%) = G_larval gain_/G_substrate_ × 100 (8)

DM_loss_ (%) = (S_i_ − LW_gain_ − Fs)/S_i_ × 100 (9)

CP_loss_ (%) = (CP_S_ − CP_larval gain_ − CP_F_)/CP_S_ × 100 (10)

G_loss_ (%) = (G_S_ − G_larval gain_ − G_F_)/G_S_ × 100 (11)

Calculation of the chemical composition parameters:

- Content of macronutrients on a dry matter basis (%):

Macronutrient % in DM = Macronutrient % in fresh medium/DM content (%) × 100(12)

- Content of individual fatty acids (FA) on dry matter basis (%):

FA (%) = FA_G_ × G/100 (13)

GR: growth rate; LW: larval weight (DM); f: final; i: initial; d: days; S: substrate (DM); Fs: frass (DM); G: total fat (DM); CP: crude protein; DM: dry matter; FA: fatty acid.

### 2.3. Statistical Analysis

Dry matter contents, larval growth and efficiency, bioconversion and chemical composition data were analyzed using two-way ANOVA, including substrate and block as fixed factors. Pairwise comparisons of substrates were performed with Tukey’s post hoc test, using the multcomp package in R [38]. Values below the detection limit were treated as missing data. For each parameter, only substrates with complete measurements were taken into account.

## 3. Results

### 3.1. Substrate Nutrient Composition

As presented in Table 2, the chemical composition and pH of the substrates were significantly different. The DM variations between the substrates were limited but they were significantly different. The CF substrate had the highest DM (39.8%) and PMLSG (31.4%) had the lowest. The crude ash varied widely between substrates, and it was by far the highest in OWF and the lowest in FFW substrates. The SW had the highest crude protein content which was 1.3 times higher than the CF protein content whereas the MS had the lowest crude protein content. The protein content of CF was not significantly different from that of FFW, but both were significantly lower than SW. Analyzing the total fat content, FFW and SW had the highest values and PMLSG and MS were below the detection level of 1%. The crude fiber content was the highest for MS (58.6%) and PMLSG (55.5%) substrates and the lowest for FFW (1.1%). Starch content was only detected in FFW and CF substrates. The control diet (CF) contained the highest number of N-free substances (63.6%), and SW and OWF contained the lowest number. Regarding the minerals, calcium was concentrated in PMS and OWF substrates that was 4.5 times higher than the calcium in CF. The lowest calcium content was recorded for the FFW substrate (0.13%). Similarly, the phosphorus content was the highest for PMS (1.5%) but the lowest for MS (0.16%) and OWF (0.19%). The pH of the substrates varied widely resulting in basic (PMS, pH = 8.35) and acidic substrates (FFW, pH = 4.79). For each chemical component, a significant difference was observed between the substrates apart from the starch content where a tendency was found.

### 3.2. Substrate Nutrient Composition Larval Growth Performance, Waste Reduction and Efficiency of Conversion

At the end of the experimental period, the containers with FFW did not contain residual substrate in addition to the frass.

**Figure 1:**
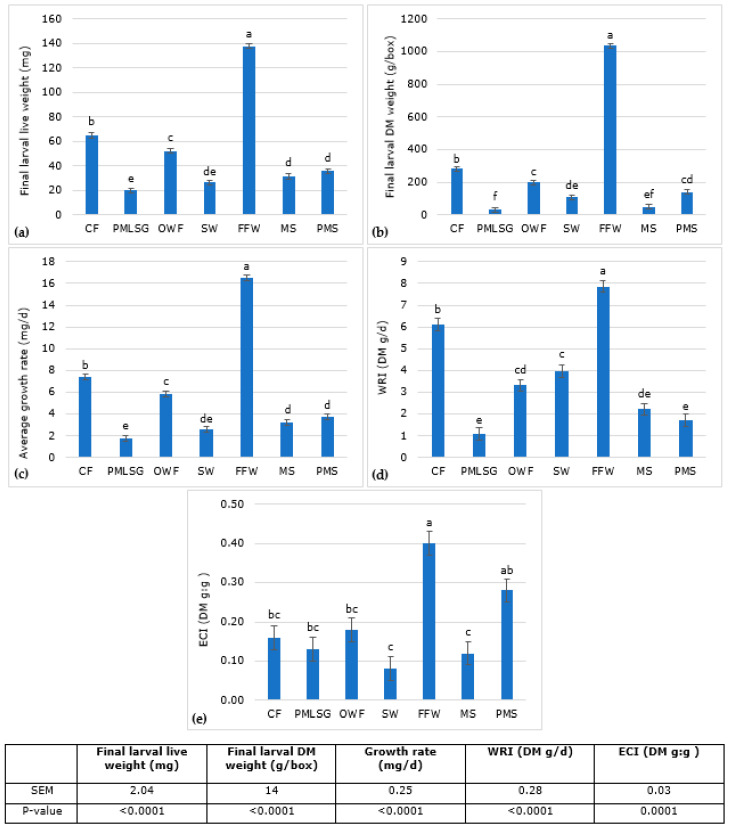
The mean values ± SEM of the final fresh (**a**) and dry (**b**) weights (mg), growth rate (mg/d) (**c**), waste reduction index (DM g/d) (**d**) and efficiency of conversion of the ingested substrate (DM g:g) (**e**) of BSFL grown on different substrates ^1^. Means with different superscript letters differ significantly (two-way ANOVA followed by Tukey’s post hoc test analysis *p* < 0.05). Chicken feed (CF; control diet), pig manure slurry mixed with roadside silage grass (PMLSG), organic wet fraction (OWF), slaughter waste (SW), fast food waste (FFW), mushroom stems (MS) and pig manure solid (PMS).

The substrates had significant effects on larval growth, waste reduction and substrate conversion (Figure 1). The highest larval growth rate was found for larvae reared on FFW (16.49 mg/d) which was 2.2 times higher than the larvae on the control substrate (CF; 7.40 mg/d). The lowest larval growth rate was observed in PMLSG (1.77 mg/d) and SW (2.60 mg/d) substrates. The same trend was observed in the Waste Reduction Index (WRI) results. The highest WRI was observed on the FFW substrate (7.86 g/d) which was 1.3 times higher than the control substrate (CF; 6.11 g/d). The WRI values decreased further for SW, OWF, MS, PMS and PMLSG substrates. WRI had similar values for SW and OWF substrates, OWF and MS substrates and MS, PMS and PMLSG substrates. The Efficiency of Conversion of the Ingested substrate (ECI) was highest on FFW (0.40) followed by PMS (0.28), which were 2.5 and 1.8 times higher than the control diet, respectively. The lowest ECI values were for MS (0.12) and SW (0.08) substrates that were similar to the control diet.

### 3.3. Larval Chemical Composition and Fatty Acid Profile

**Figure 2:**
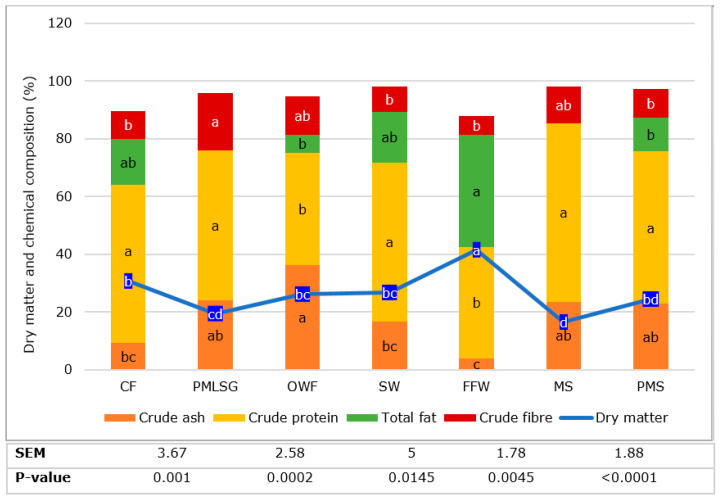
The mean values of the dry matter and the chemical composition of the larvae reared on different substrates ^1^ (macronutrients expressed in % of dry matter). For each nutrient, means with different superscript letters differ significantly (two-way ANOVA followed by Tukey’s post hoc test analysis *p* < 0.05). Chicken feed (CF; control diet), pig manure slurry mixed with roadside silage grass (PMLSG), organic wet fraction (OWF), slaughter waste (SW), fast food waste (FFW), mushroom stems (MS) and pig manure solid (PMS).

The macronutrient chemical composition of the larvae was analyzed and the results are presented in Figure 2. Significant differences were found between substrate groups for all of the macronutrients. Larvae reared on the FFW had the highest dry matter content (41.50%) which was 1.4 times higher than the control diet and the lowest was observed on MS substrate. Crude ash content of the larvae was the highest on OWF and the lowest on FFW substrates. Based on dry matter, the larval crude protein content was the highest on MS, SW, CF, PMS and PMLSG substrates compared to FFW and OWF substrates. The FFW and OWF substrates had 1.4 times lower crude protein compared to the CF control diet. Total fat content was the highest in larvae reared on FFW, SW and CF substrates. Larval fat content in PMLSG and MS was below the detection level of 1% which was the lowest. The larval crude fiber content was the highest for PMLSG and the lowest for FFW. The N-free substance content was the highest on FFW (12.2%).

The fatty acid contents and profile of the larvae were analyzed and the results are presented in Table 3 and in Table S1. Substrates had significant effects on the fatty acid content of larvae. In general, larvae reared on FFW had the highest concentration of different fatty acids and the lowest when reared on OWF according to the total fat content. The larvae reared on PMLSG and MS substrates had undetectable values of fatty acids. The differences in the larval content of capric acid and lauric acid between different substrates were very high. Both were 32 times higher in larvae reared on FFW compared to OWF and 2.5 times higher compared to the chicken feed control diet. However, the differences in the content of other fatty acids across the substrates were less. Myristic acid was 11.2 times higher in FFW compared to OWF and 1.8 times compared to the control which was not significantly different. The sums of saturated and monounsaturated fatty acids and omega-6 fatty acids were the highest on the FFW substrates and the lowest on OWF. The sum of polyunsaturated fatty acid was not significantly different but tendency for the substrate’s effect was found. The sum of trans and omega-3 fatty acids content was not significantly different between substrates.

### 3.4. Frass Chemical Composition

The different substrates affected the chemical composition of frass (Table 4). There were vast differences in the content of crude ash, crude protein, total fat, crude fiber, calcium and phosphorus in the frass, while the differences were limited in the content of DM and N-free substances. The highest dry matter was recorded for the frass resulting from the larvae reared on FFW and the control diet and the lowest in OWF frass. The OWF frass had the highest crude ash content (39.8%) while the lowest crude ash was measured in MS frass (3.8%). The highest crude protein content was found in SW frass that was 1.3 times higher than CF and the lowest in MS frass that was 4.6 times lower than the crude protein content in CF frass. Frass produced from FFW had the highest amount of total fat which was 9.2 times higher than the total fat in the CF frass. The lowest total fat amounts were below the detection limit in PMLSG, MS and PMS frass. The crude fiber content of the frass was the highest when the larvae were reared on the MS substrate and the lowest on FFW. The N-free substance content ranged between 38.9% in CF frass and 16.2% in OWF frass. The highest calcium content was measured in PMS frass and the lowest in FFW frass. The phosphorus was the highest in PMS frass and the lowest in MS and OWF frass.

In general, recordings of the pH indicated neutral to basic frass. Rearing insects on PMS and PMLSG resulted in basic frass with a pH close to 9; however, the pH of frass in FFW treatment was slightly acidic (pH = 6.8). The pH of frass in the rest of the treatment was neutral or slightly basic: between 7.4 and 8.

### 3.5. Larval Crude Protein and Fat Masses, Conversions and Losses

**Figure 3:**
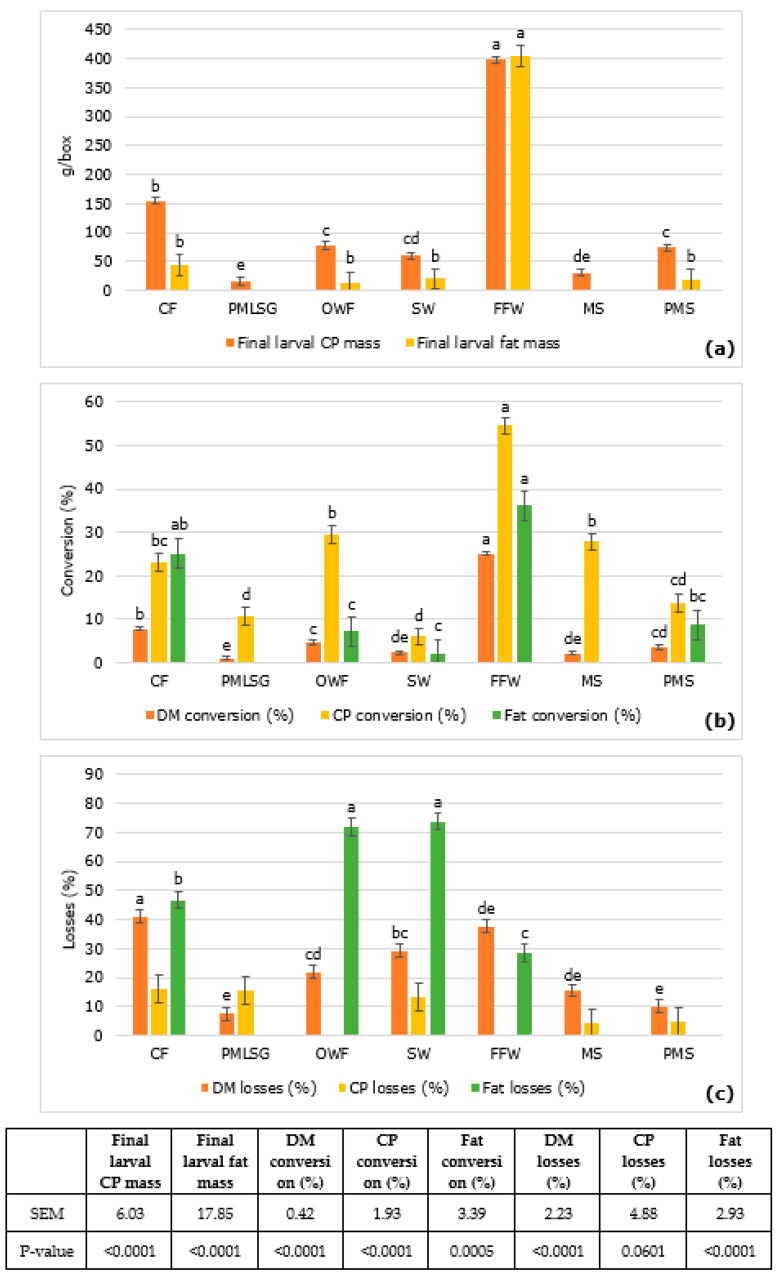
The mean values ± SEM of the crude protein and fat masses (g/box) (**a**), conversions (%) (**b**) and losses (%) (**c**) of larvae reared on different substrates ^1^ (based on dry matter). Means with different letters in a row are significantly different (two-way ANOVA followed by Tukey’s post hoc test analysis *p* < 0.05). Chicken feed (CF; control diet), pig manure slurry mixed with roadside silage grass (PMLSG), organic wet fraction (OWF), slaughter waste (SW), fast food waste (FFW), mushroom stems (MS) and pig manure solid (PMS). Missing data were below the limit of detection.

Larval crude protein and fat masses, conversions and losses based on the dry matter are presented in Figure 3. The different substrates significantly affected the mentioned parameters, apart from the crude protein loss, where a tendency for substrate effect was found. In general, the FFW substrate had the best performance regarding the CP and fat masses and conversions.

Larvae reared on FFW contained the highest crude protein mass (398.4 g/box), which is 2.6 times higher than the control diet (CF; 154.3 g/box) and 25.5 times higher than PMLSG which contained the lowest larval crude protein mass (15.6 g/box). Total larval fat mass was significantly higher when FFW substrate (404.3 g/box) was applied compared to all other substrates. It was 9.1 times higher compared to the control diet (CF 44.62 g/box). In PMLSG and MS, the total larval fat content was below the detection level of 1%.

Following the same trend, FFW had the highest values of DM, CP and fat conversion with respect to other diets. DM conversion on FFW substrate (25.2%) was 3.2 times higher than CF (7.89%) and the lowest was obtained on PMLSG (1.2%). Crude protein conversion of the FFW substrate (54.5%) was 2.4 times higher than that of CF (23.14%) and the lowest conversion values were for SW (6.1%) and PMLSG (10.75%) substrates. Fat conversion on FFW (36.2%) was not significantly different from the CF control diet. SW had the lowest fat conversion rate (2.03%).

The CF control substrate had the highest dry matter loss (41.0%) that was similar to the FFW substate. The lowest dry matter loss was observed in PMLSG that was 5.5 times less than CF and PMS substrates. Reared on the SW substrate, the larvae had the highest fat loss (73.7%) which was similar to OWF (71.9%) compared to other substrates. FFW had the lowest value of fat loss but PMLSG, MS and PMS values were below the detection level of 1%.

## 4. Discussion

Rearing BSFL on different substrates affects the larval DM which was between 16.4 and 41.5% among substrates. The highest larval DM was observed in FFW substrate (41.5%) which was 1.4 times higher than the larval DM of the CF control diet. Several factors may affect larval DM accumulation such as the larval fat, the substrate composition and the larval stage. The fat content of larvae reared on FFW was the highest and this leads to low water accumulation in the larvae and thus higher larval DM. This trend applies to other substrates in which the larval DM and fat are positively correlated. This reasoning was also mentioned by Eriksen [39]. Similarly, in a previous study, the larval DM was proportional to the larval fat content [29]. On the other hand, in our study, as well as in the study of Veldkamp [33], the substrates are composed of widely different products (organic waste of both plant and animal origins and agro-industrial by-products) and the larval DM showed a wide range of variability. The effect of the substrate on the larval DM needs to be further studied because the literature is contradictory in this regard [15,33]. In addition, Liu [40] in their study proved that larval DM fluctuates during different lifecycle stages of BSF. The larval DM increased between different larval instars and reached a maximum at the last larval instar before it declined during the prepupal and pupal stages [40]. This finding may explain the reason behind the high larval DM on FFW in the current study. The larvae reared on FFW substrate developed well (highest growth rate and fresh weight) with higher larval instars compared to other substrates and thus more DM content was accumulated.

In this study, larvae reared on FFW substrate demonstrated the best larval performance and composition. They showed the highest values for dry matter (41.5%), final weight (137.8 mg), WRI (7.9 g/d), ECI (0.4) and total fat (39.0%). Moreover, they had the highest crude protein (CP) mass (398.4 g/box), fat mass (404.3 g/box), DM conversion (25.2%), CP conversion (54.5%) and fat conversion (36.2%). However, the CP content of the larvae reared on FFW was the lowest of all substrates (38.5%). These results partially agree with the results of Nguyen [41]. In the later study, kitchen waste was collected from restaurants and contained both animal and plant matter (hamburgers and salads) and had a 20.41% protein content. BSFL reared on these types of waste had the significantly highest larval weight and larval length but also the highest protein content (21.2%) compared to other experimental substrates [41] unlike our case for CP. In fact, in the current study, the larval CP was between 38.5 and 61.9% for FFW and MS substrates, respectively. Although rearing larvae on FFW resulted in the lowest CP content in our study, the value was still higher than the highest value (21.2%) in Nguyen [41]. This result may have many reasons to explain. The CP content of the substrate may not necessarily determine the larval CP. Furthermore, the presence of chitin and other non-protein nitrogen-containing compounds (e.g., nucleic acids, uric acid, urea, and ammonia) may result in an overestimation of the crude protein content in BSF when using the standard nitrogen-to-protein conversion factor of 6.25 [42] and FFW probably had more non-protein nitrogen so the larvae could not store protein.

Additionally, the high fat composition of FFW may have prevented the deposition of CP in larvae. Indeed, the case of the MS substrate that had the lowest CP content but resulted in the highest larval CP may support the hypothesis. These uncertainties can be addressed in further studies on the effects of greasy fast food waste substrates on larval protein content. In addition, the high number of the starting larvae in the FFW container may explain the reason behind the low larval CP content (38.50%) but the highest larval CP mass harvested per container (398.39 g/box).

The nutritional composition of the substrates affects the growth performance and the chemical composition of BSFL remarkably. The larvae reared on MS showed the worst growth and development and the poorest composition in this study, followed by those reared on PMLSG substrates. In other words, neither substrates lead to favorable results of several studied parameters, such as larval dry matter and final dry mass, total fat, larval protein and fat masses, dry matter and fat conversions and dry matter and fat losses. In comparison to other substrates in this study, both of these substrates had a high crude fiber content (MS: 58.62% and PMLSG: 55.54%), a low crude protein content (MS: 6.18%, PMLSG: 8.22%) and no starch and fat contents. However, the larvae reared on these substrates had a high CP content. In fact, the highest content of CP in larvae was observed in MS treatment (61.9%) and the larval CP content was not significantly different in PMLSG treatment. Previous studies evaluating the effect of rearing BSFL on low protein substrates observed similar results indicating the ability of the BSFL in converting low-quality waste into rich protein products. However, this ability should be further studied and optimized as other growth parameters are also relevant for a commercially successful production process. Similar results were presented by Gold [43] in which mill by-product substrates with low protein (14.5%) and fat (3.0%) contents resulted in larvae with a high protein content (42.1%) and a high protein conversion efficiency. In addition, in the study of Fischer and Romano [44], BSFL had high protein content (33–42%) despite the fact that all of the tested substrates (fruits, vegetables and starches) were relatively low in protein (4.06–19.26%).

In contrast, rearing larvae on another low CP substrate in this study, OWF (CP = 6.87%), resulted in the lowest larval CP content. Substrates with similar nitrogen content do not necessarily contain similar crude protein contents or similar amino acid contents which are utilizable for BSFL. Therefore, more studies are needed to elaborate the role of crude protein intake, or the intake of different amino acids by BSFL on the growth performance and nutritional composition of larvae.

Without denying the abilities of BSFL to transform the low-quality waste products into high-quality product, there is yet another possible explanation for the high protein content of larvae reared on MS in our study: the low growth performance of BSFL in this treatment since all the treatments were harvested in the same day. The growth rate value and the fresh and dry weights of the larvae reared on MS and PMLSG were lower than the control diet. The lower larval instars usually have higher protein concentrations. This was confirmed by Liu [40] who studied the nutrient composition of BSF from eggs until the adult stage and confirmed our results. Their results showed that among the larval instars (larval stage) reared on the same substrate (commercial broiler chicken feed), the 1-day-old larvae had the highest protein content (56.2%) which decreased throughout the larval instars to reach the lowest values of 38.0% for the 12-day-old larvae and 39.2% for the 14-day-old larvae. However, the prepupae (last larval instar before pupation) had a 40% crude protein content [40]. Indeed, this explains the reason behind the high protein content in the less developed larvae (reared on MS). Compared to MS substrates, OWF is also a low CP substrate but resulted in low larval CP content. Reread on OWF, the larval growth rate and final fresh and dry weights were higher than that of MS which led to less protein accumulation because higher larval instars have a lower protein concentration [40]. Therefore, the growth and the development level of the larvae may also affect their CP accumulation. In addition, Eggink [42] concluded that the larval composition is affected by the rearing substrate but mostly due to differences in larval development, as reflected in the growth rate, rather than directly reflecting the substrate composition. However, this conclusion is not yet studied enough and only Liu [40] has tackled this topic.

In the literature, substrates rich in fibers showed positive impacts on the larval composition of BSFL. The larval CP content was high when high fiber content substrates were used [15,31,45]. These results confirm the results of MS and PMLSG substrates in this study that have the highest fiber contents and the highest larval CP. However, larvae reared on PMLSG had the lowest larval growth rate (1.77 mg/d) and the correlation between the fiber content and the larval growth rate was negative. This is in accordance with the results of Liu [46] in which the fiber-rich substrate had the lowest larval growth rate. Besides, the results of Ramzy [47] showed that BSFL reared on fiber-rich (lignin) substrates had the significantly lowest protein content compared to other substrates of medium and low fibre contents. Fibers and specifically lignin are barely degradable by BSFL [46,47,48]. Moreover, the analyzed fiber content based on different methods may differ between substrates (dietary fiber, crude fiber, lignin, NSP-soluble and insoluble, acid detergent lignin, etc.). Furthermore, other characteristics of fiber content in substrates may affect its fate (particle size, soluble or insoluble, viscosity, water holding capacity, etc.). Therefore, fiber is a complicated component, and its effect on the growth and the composition of BSFL should be studied more in detail to settle this controversial debate.

BSF is rich in saturated fatty acids and especially lauric acid that distinguishes BSF from other insects. It is known for its antimicrobial activity against gram-positive bacteria [49], different pathogens and antimicrobial peptide-resistant bacteria [50]. In this study, lauric acid content was the highest (13.44%) along with the total fat content when the larvae were reared on FFW. The high dietary fat leads to high larval fat that could explain the higher synthesis of fatty acids by utilizing dietary fatty acids, mainly saturated fatty acids as lauric acid [49].

Additionally, FFW had the lowest fiber content (1.13%; 5.5 times less than the CF control diet and 51.9 times less than MS, the highest fiber content substrate) and the highest protein content compared to other substrates. The lauric acid content in BSFL reared on FFW was 13.44 which is 2.5 times more than the control and 11 times more than MS. Similarly in Fischer [51], the larvae reared on low cellulose content substrates had the significantly highest lauric acid level (158 mg/g vs. 66.44 mg/g). Others similarly found that lauric acid in BSFL was lower in substrates with a higher content of indigestible fibers [14,52], which supports our findings. The presence of fiber may affect the feeding patterns of BSFL and may lead to reduction in feed-intake and thus less deposition of fat and fatty acids. However, contradictory results were presented by Ramzy [47] in which the larvae reared on low-fiber substrates (low lignin) had the lowest lauric acid content (54.24%) compared to medium and rich fiber substrates. Therefore, the relationship between the fiber content of the substrate and lauric acid deposition in larvae is contradictory and should be studied in more detail also considering the fatty acid profile of the substrate for a better comparison.

Growth rate is a useful performance parameter that was tackled in different studies [15,46]. In our study, the highest average growth rate was observed for larvae reared on FFW (16.48 mg/day) and lowest was observed when reared on PMLSG (1.77 mg/d) which was 2.2 times higher and 4.2 times lower than the growth rate in the control substrate, respectively. In Meneguz’s [15] study, the larval growth rate ranges between 6 mg/day when reared on organic matter (vegetables and fruits) or winery wastes and 14 mg/day when reared on brewery wastes. The CP content of the diets used by Meneguz ranged between 4.6 and 12% for organic waste and between 11.7 and 20.1% for agro-industrial by-products. However, the fat content was about 2.6% in organic waste and ranged between 8 and 8.7% for agro-industrial by-products [15]. These values were comparable to our results. Additionally, when reared on pig manure in the study of Liu [46], the larval growth rate was the second lowest (0.8 mg/d) and the highest larval growth rate (2.8 mg/day) was for the chicken feed control diet. Therefore, in this study as in the mentioned studies [15,46], the growth rate of BSFL follows the same trend as the final body weight and the DM larval mass, and thus the rich diet from protein and fat perspectives leads to higher larval growth.

The waste reduction index (WRI) is an important variable studied in different experiments. In this study, the WRI was the highest for FFW (7.86 g/d) followed by CF (6.11 g/d), and the lowest reduction index was measured for PMLSG (1.08 g/d) and PMS (1.71 g/d). Similar results were presented by Veldkamp [33] where the larvae reared on catering swill waste achieved the highest WRI (10.6 g/d) and pig manure solid substrate showed a low value of WRI (3 g/d). Additionally, in another study the larvae reared on cow manure had the lowest WRI (12.7%), and the highest WRI was achieved by larvae reared on poultry feed (67.7%) [43]. The conclusion may be that pig manure was not the preferred attractive substrate for BSFL and it was consumed for surviving reasons only. The reasons may be the low palatability of pig manure or the low availability of nutrients. When pig manure was mixed with chicken feed in Veldkamp [33], the WRI increased significantly (5.4 g/d) which confirms the mentioned conclusion.

Looking at the protein conversion efficiency, larvae reared on FFW had the highest value (54.54%), 2.4 times higher than the control diet, and the lowest was for SW (6.10%). In a previous study, Gold [43] showed that the canteen waste (containing meat and vegetables) led to a high protein conversion efficiency that was statistically similar to the highest value of protein conversion efficiency of the mill by-products substrate. Therefore, although the larval crude protein was the lowest when reared on fast food waste, the larvae were efficiently transforming their inputs into animal protein.

However, in addition to the nutritional composition of substrates, other factors such as the free water and the particle size of the substrate may affect the BSF larval growth. The free water available in the substrate may lead the larvae to escape early before developing into the prepupal stage. The free water is a crucial factor that has impacts on larval growth and development, as mentioned by Bekker [53]. The later study concluded that the substrate moisture content affects the microbial activity in the substrate, the length of the larval growth phase and the larval size. In the current study, the substrates’ moisture content is 65% but the water-holding capacity (WHC) of each substrate was not measured which led to water–substrate stratification. On the other hand, Naser El Deen [54] studied the effect of substrate particle size on the larval growth of *Tenebrio molitor*. The results showed that different particle sizes affected the growth rate and performance of mealworms in which a particle size smaller than 2 mm was preferred by the larvae. In the current study, the substrates were shredded into different particle sizes based on the type of each substrate and this may have affected the BSFL growth. Theoretically, the ideal particle size for BSFL is around 0.15 mm [55] which is not practical. Therefore, further studies should be conducted to determine the best particle size for BSFL feed.

Frass, as a second end-product of BSFL rearing, includes insect (larval) excrements, exoskeleton sheds and remaining feeding substrate. A substantial amount of frass is left behind and if not used correctly may form an environmental burden. So far, frass seems to be suitable to use as soil fertilizer or amendment [56]. The chemical composition of substrates affected the chemical profile of frass in this study which is in accordance with the literature available [44,57,58]. Larvae reared on FFW produced frass with the highest DM (78.0%) and the lowest value was found for OWF (45.64%). Unlike the results of another study where the DM of frass was not significantly different between different larval substrates (chicken feed, fruit/vegetable mix and grass-cuttings) [57]. However, the DM of frass is not a solidate parameter to compare different frass qualities because it may vary widely based on several factors such as the DM of the substrate, the ratio of larvae to substrate in the experimental setup, the temperature and the relative humidity. Therefore, even when the same substrates are used, the resulting DM of frass may differ when different experimental conditions are applied and this should be further investigated to draw a clear conclusion on the effect of each factor on the frass quality and composition.

Crude protein and total fat contents of the frass reflected the crude protein and fat contents of the substrates in this study. Frass with the highest (31.44%) and lowest protein (5.07%) contents was produced by larvae reared on substrates with the highest (SW; 26.6%) and lowest (MS; 6.18%) protein contents, respectively. Similarly, the total fat content in frass was highest (26.45%) when larvae were reared on FFW and lowest (1.24%) when reared on OWF. Both mentioned substrates had the highest and the lowest fat contents, respectively (27.74% and 4.39%, respectively). However, the frass resulting from SW and FFW substates had a different fat content although the fat content in these substrates was similar. Therefore, the claimed positive correlation between the chemical composition of the substrates and frass is not always correct and cannot be generalized. To the knowledge of the authors, the nutritional compositions (ash, protein and fat) of frasses resulting from different experimental substrates were not analyzed and compared in other studies which makes the discussion very limited. Most of the studies concentrate on analyzing the chemical elements in the frass such as nitrogen, phosphorus and potassium (NPK) and other micronutrients to check the frass’s suitability as a soil fertilizer or amendment [44,51,59,60,61]. It was also demonstrated that the type of substrate significantly impacted the NPK values of the frass [44].

In this study, calcium and phosphorus contents in the frass were proportional to the substrate calcium and phosphorus contents. Frass with the highest calcium (3.30%) and phosphorus (1.71%) was produced from the PMS substrate that has the highest calcium and phosphorus composition. Similarly, FFW with the lowest calcium resulted in low calcium frass (0.07%), and MS and OWF substrates with the lowest phosphorus resulted in low phosphorus frass (0.17% and 0.18%, respectively). The highest calcium and phosphorus contents were measured in frass from larvae reared on PMS and this can be explained by the fact that pig manure is a rich source of phosphorus and calcium [62]. In Fischer [51], frass from spent coffee had the lowest phosphorus content like the substrate and this approves our results.

The main hurdle that prohibits formulating a firm conclusion about the frass chemical composition is the fact that the frass is mixed with the unconsumed substrate so the composition of the frass may not be highly reliable and representative.

## 5. Conclusions

The results of this study showed that the BSFL were able to exploit and survive on a wide range of waste streams, organic waste of both plant and animal origins and agro-industrial by-products. However, this does not mean that BSFL will perform the same on all substrates. In this study, BSFL had the highest growth performance and development when reared on fast food waste which led to the highest quantity of harvested larvae. However, larvae reared on pig manure slurry mixed with roadside silage grass (PMLSG) performed worst for some parameters on mushroom stems (MS). In addition, frass chemical composition varied based on substrates, which proves that substrates do not only impact the larvae . This experiment leads to two very important conclusions. (1) The ability of the black soldier fly larvae to grow and survive on very low-quality substrates such as PMLSG and MS, and transform them into high-quality material rich in protein and fat. Mushroom stems and roadside silage grass are not well-researched substrates that deserve attention due to their high volumes. Likewise, pig manure and sludge from slaughter waste are critical materials that should be extensively explored for future opportunities as BSF feeding substrates. (2) Substrate composition plays a crucial role in every parameter of BSF rearing including growth, development and chemical composition of the larvae in addition to the chemical composition of the frass.

## Figures and Tables

**Table 1:** An overview of the source of each substrate, the quantity of the wet substrate (kg) and the number of larvae at the beginning of the trial in each repetition (container) across the treatments.

Substrate	Source	Quantity of Wet Substrate (kg)	Number of BSFL
Chicken feed (CF; control diet)	Agruniek Rijnvallei Voer BV (Wageningen, The Netherlands)	8.0	14,800
Pig manure slurry mixed with roadside silage grass (PMLSG)	Van Beek SPF Varkens B.V. (Lelystad, The Netherlands)	5.0	9250
Organic wet fraction (OWF)	Attero Holding N.V. (Arnhem, The Netherlands)	10.0	18,500
Secondary sludge from slaughter waste (SW)	Esbro (Doetinchem, The Netherlands)	10.0	18,500
Fast food waste (FFW)	McDonald’s restaurants (The Netherlands)	10.0	18,500
Mushroom stems (MS)	CNC Grondstoffen BV (Milsbeek, The Netherlands)	5.0	9250
Pig manure solid (PMS)	Van Beek SPF Varkens B.V. (Lelystad, The Netherlands)	9.0	16,650

**Table 2:** Chemical composition and pH of the substrates ^1^ (dry matter expressed in %, all macronutrients expressed in % of dry matter).

Parameter	CF	PMLSG	OWF	SW	FFW	MS	PMS	SEM	*p*-Value
Dry matter	39.80 ^a^	31.40 ^c^	35.77 ^ac^	34.33 ^bc^	38.33 ^ab^	32.97 ^c^	35.73 ^ac^	0.98	0.00075
Crude ash	5.19 ^c^	5.45 ^c^	37.79 ^a^	3.49 ^c^	3.13 ^c^	3.44 ^c^	15.40 ^b^	0.80	<0.0001
Crude protein	19.77 ^b^	8.22 ^d^	6.87 ^d^	26.60 ^a^	18.09 ^b^	6.18 ^d^	15.86 ^c^	0.42	<0.0001
Total fat	5.28 ^bc^	-	4.39 ^c^	27.38 ^a^	27.74 ^a^	-	5.98 ^b^	0.22	<0.0001
Crude fiber	6.20 ^c^	55.54 ^a^	29.30 ^b^	26.50 ^b^	1.13 ^d^	58.62 ^a^	26.02 ^b^	0.81	<0.0001
Starch	42.88	-	-	-	45.92	-	-	0.66	0.08336
N-free substances	63.57 ^a^	30.80 ^c^	21.66 ^d^	16.02 ^d^	49.91 ^b^	31.77 ^c^	36.74 ^c^	1.61	<0.0001
Calcium	0.70 ^b^	0.74 ^b^	3.12 ^a^	0.89 ^b^	0.13 ^c^	0.71 ^b^	3.13 ^a^	0.08	<0.0001
Phosphorus	0.64 ^b^	0.31 ^cd^	0.19 ^e^	0.40 ^c^	0.24 ^de^	0.16 ^e^	1.50 ^a^	0.02	<0.0001
pH	5.26 ^e^	7.69 ^b^	5.79 ^c^	5.67 ^cd^	4.79 ^f^	5.38 ^de^	8.35 ^a^	0.08	<0.0001

Means with different superscript letters differ significantly (two-way ANOVA followed by Tukey’s post hoc test analysis *p* < 0.05). Chicken feed (CF; control diet), pig manure slurry mixed with roadside silage grass (PMLSG), organic wet fraction (OWF), slaughter waste (SW), fast food waste (FFW), mushroom stems (MS) and pig manure solid (PMS). (-) below the limit of detection.

**Table 3:** Fatty acid profile of the larvae reared on different substrates ^1^ (fatty acids expressed in % of dry matter).

Parameter	CF	PMLSG	OWF	SW	FFW	MS	PMS	SEM	*p*-Value
Capric acid C 10:0	0.13 ^b^	-	0.01 ^b^	0.08 ^b^	0.32 ^a^	-	0.04 ^b^	0.03	0.0002
Lauric acid C 12:0	5.40 ^b^	-	0.42 ^c^	2.05 ^bc^	13.44 ^a^	-	1.23 ^c^	0.83	<0.0001
Myristic acid C 14:0	1.36 ^ab^	-	0.22 ^c^	0.72 ^bc^	2.46 ^a^	-	0.45 ^bc^	0.23	0.0009
Palmitic acid C 16:0	2.17	-	1.64	4.57	4.94	-	3.02	1.23	0.3222
Sum saturated fatty acids	9.72 ^b^	-	2.65 ^b^	8.32 ^b^	22.49 ^a^	-	5.45 ^b^	2.42	0.0034
Sum monounsaturated fatty acids	3.16 ^ab^	-	2.40 ^b^	6.42 ^ab^	11.04 ^a^	-	4.38 ^ab^	1.73	0.0459
Sum polyunsaturated fatty acids	2.99	-	1.28	3.04	5.41	-	1.97	0.85	0.0674
Sum trans fatty acids	0.12	-	0.05	0.22	0.26	-	0.15	0.05	0.1028
Omega-3 fatty acids	0.25	-	0.14	0.28	0.42	-	0.15	0.08	0.1711
Omega-6 fatty acids	2.66 ^ab^	-	1.12 ^b^	2.67 ^ab^	4.86 ^a^	-	1.77 ^ab^	0.75	0.0609

Means with different superscript letters differ significantly (two-way ANOVA followed by Tukey’s post hoc test analysis *p* < 0.05). Chicken feed (CF; control diet), pig manure slurry mixed with roadside silage grass (PMLSG), organic wet fraction (OWF), slaughter waste (SW), fast food waste (FFW), mushroom stems (MS) andpig manure solid (PMS). (-) below the limit of detection.

**Table 4:** Chemical composition of the frass by substrates ^1^ (dry matter expressed in %, all macronutrients expressed in % of dry matter).

Parameter	CF	PMLSG	OWF	SW	FFW	MS	PMS	SEM	*p*-Value
Dry matter	71.90 ^a^	53.87 ^c^	45.64 ^d^	59.33 ^bc^	78.00 ^a^	63.03 ^b^	55.87 ^bc^	1.49	<0.0001
Crude ash	7.80 ^c^	5.64 ^cd^	39.82 ^a^	4.55 ^cd^	6.28 ^cd^	3.75 ^d^	16.82 ^b^	0.66	<0.0001
Crude protein	23.57 ^b^	6.57 ^d^	6.96 ^d^	31.44 ^a^	22.73 ^b^	5.07 ^d^	14.98 ^c^	0.43	<0.0001
Total fat	2.86 ^c^	-	1.24 ^c^	9.79 ^b^	26.45 ^a^	-	-	0.66	<0.0001
Crude fiber	26.92 ^d^	61.07 ^b^	35.75 ^c^	35.14 ^c^	10.98 ^e^	67.59 ^a^	32.56 ^c^	0.90	<0.0001
N-free substances	38.85 ^a^	26.73 ^b^	16.23 ^d^	19.07 ^cd^	33.56 ^a^	23.58 ^bc^	35.04 ^a^	1.27	<0.0001
Calcium	0.81 ^bc^	0.65 ^bc^	2.30 ^ab^	1.06 ^bc^	0.07 ^c^	0.55 ^bc^	3.30 ^a^	0.38	0.0007
Phosphorus	0.96 ^b^	0.30 ^d^	0.18 ^e^	0.49 ^c^	0.34 ^d^	0.17 ^e^	1.71 ^a^	0.02	<0.0001
pH	8.01 ^abc^	8.72 ^ab^	7.34 ^cd^	7.67 ^bd^	6.81 ^d^	7.96 ^abc^	8.87 ^a^	0.22	0.0003

Means with different superscript letters differ significantly (two-way ANOVA followed by Tukey’s post hoc test analysis *p* < 0.05). Chicken feed (CF; control diet), pig manure slurry mixed with roadside silage grass (PMLSG), organic wet fraction (OWF), slaughter waste (SW), fast food waste (FFW), mushroom stems (MS) and pig manure solid (PMS). (-) below the limit of detection.

## Data Availability

Data are available upon request from the corresponding author and pending agreement by co-authors.

## Supplementary Materials

The following supporting information can be downloaded at: https://www.mdpi.com/article/10.3390/insects14020204/s1, Table S1: Fatty acid profile of the larvae reared on different substrates ^1^ (all fatty acids expressed in % of dry matter).
