# Supplementary material for: Bioconversion of Different Waste Streams of Animal and Vegetal Origin and Manure by Black Soldier Fly Larvae Hermetia illucens L. (Diptera: Stratiomyidae)
Source: Insects. 2023 Feb 17;14(2):204. doi: 10.3390/insects14020204 (PMC9968099; doi:10.3390/insects14020204)
Supplement: Supplementary file 1 [file insects-14-00204-s001.zip › insects-2206091-supplementary.pdf]

Table S1. Fatty acid profile of the larvae reared on different substrates [1] (all fatty acids expressed in % of dry matter).

Parameter	CF	PMLSG	OWF	SW	FFW	MS	PMS	SEM	P-value
Myristoleic acid C 14:1	0.03^{b}	-	0.02^{b}	0.06^{ab}	0.12^{a}	-	0.06^{ab}	0.01	0.0036
Pentadecanoic acid C 15:0	0.03	-	0.03	0.07	0.08	-	0.08	0.01	0.0411
Hexadecanoic acid trans-isomers C 16:1 trans	-	-	-	0.02	-	-	0.02	0.002	0.7476
Palmitoleinic acid C 16:1	0.30	-	0.16	1.20	0.77	-	0.80	0.35	0.2974
Margaric acid C 17:0	0.06^{ab}	-	0.03^{b}	0.05^{ab}	0.08^{a}	-	0.06^{ab}	0.005	0.0060
Stearic acid C 18:0	0.54^{ab}	-	0.27^{b}	0.76^{ab}	1.08^{a}	-	0.55^{ab}	0.16	0.0633
Octadecenoic acid trans-isomers C 18:1 trans	0.04	-	0.03	0.11	0.13	-	0.09	0.02	0.0667
Oleic acid C 18:1	2.28^{b}	-	2.04^{b}	4.66^{ab}	9.51^{a}	-	3.17^{b}	1.26	0.0164
Cis-vaccenic acid C 18:1	0.10	-	0.04	0.19	0.21	-	0.12	0.05	0.2816
Octadecadienoic acid trans-isomers C 18:2 trans	0.03^{ab}	-	-	-	0.08^{a}	-	0.02^{b}	0.01	0.0333
Linolic acid C 18:2 omega-6	2.64^{ab}	-	1.09^{b}	2.61^{ab}	4.84^{a}	-	1.72^{ab}	0.73	0.0537
Octadecatetrienic acid, trans-isomers C 18:3 trans	0.04	-	-	-	0.05	-	-	0.02	0.8348
Alpha-linolenic acid C 18:3 omega-3	0.25	-	0.11	0.20	0.39	-	0.13	0.06	0.0684
Arachic acid C 20:0	0.02^{b}	-	0.01^{b}	0.02^{b}	0.05^{a}	-	0.02^{b}	0.005	0.0060
Eicosenoic acid C 20:1	0.40^{a}	-	0.11^{b}	0.16^{b}	0.31^{ab}	-	0.12^{b}	0.05	0.0073
Arachidonic acid C 20:4 omega-6	-	-	0.02	-	-	-	0.04	0.02	0.5682
Eicosapentaenic acid C 20:5 omega-3	-	-	0.03	0.04	-	-	0.02	0.01	0.4256

Means with different superscript letters differ significantly (two-way ANOVA followed by Tukey's post hoc test analysis $p<0.05$). [1] Chicken feed (CF; control diet), Pig manure slurry mixed with roadside silage grass (PMLSG), Organic wet fraction (OWF), Slaughter waste (SW), Fastfood waste (FFW), Mushroom stems (MS), Pig manure solid (PMS). (-) below the limit of detection
